# The Secure Base in the Storm: How Parent–Child Bonds Shape Coping in Pediatric Cancer Caregiving

**DOI:** 10.3390/pediatric18020052

**Published:** 2026-04-02

**Authors:** Damiano Rizzi, Lavinia Barone, Alessandra Balestra, Maria Montanaro, Francesca Nichelli, Emanuela Schivalocchi, Giulia Rampoldi, Marco Spinelli, Giulia Ciuffo, Letizia Pomponia Brescia, Valerio Cecinati, Marco Zecca, Claudia Greco, Francesca Lionetti, Jessica Rotella, Giulia Gambini, Catherine Klersy, Chiara Ionio

**Affiliations:** 1 Department of Brain and Behavioral Sciences, University of Pavia, 27100 Pavia, Italy; 2 Soleterre ETS, Viale Cassinis 7, 20139 Milan, Italy; 3 Trauma Research Unit, Department of Psychology, Catholic University of the Sacred Heart, Largo Gemelli 1, 20123 Milan, Italy; 4 Fondazione Monza e Brianza per il Bambino e la sua Mamma c/o Irccs San Gerardo Monza, via Pergolesi 33, 20900 Monza, Italy; 5 Fondazione Irccs San Gerardo dei Tintori, S. C. Pediatria, via Pergolesi 33, 20900 Monza, Italy; 6 SC di Pediatria/Oncoematologia Pediatrica, Ospedale SS Annunziata, Via Bruno, 1, 74121 Taranto, Italy; 7 SC Ematologia 2–Oncoematologia Pediatrica, Fondazione IRCCS Policlinico San Matteo, Viale Golgi 19, 27100 Pavia, Italy; 8 BERD Unit, Direzione Scientifica, Fondazione IRCCS Policlinico San Matteo, Viale Golgi 19, 27100 Pavia, Italy

**Keywords:** paediatric oncology, coping, attachment, caregivers, psychological adjustment, parent–child relationship, resilience

## Abstract

Background: A paediatric cancer diagnosis is a profound stressor for the entire family system. Although coping strategies are well-studied, their link to the quality of the parent–child attachment relationship remains less explored. In this study, we investigated whether dyadic attachment dynamics—specifically closeness and conflict between parent and child—are associated with the use of adaptive or maladaptive coping strategies in caregivers of children undergoing active treatment for oncohaematological diseases. Methods: We conducted a multicentre, cross-sectional study across three Italian paediatric oncohaematology centres. A total of 165 caregivers of 91 paediatric patients aged 3–17 years completed self-report measures assessing parent–child relationship quality (Child–Parent Relationship Scale-CPRS), coping strategies (COPE-NVI), perceived social support (MSPSS), and resilience (RS-14). We tested whether the quality of the parent–child attachment relationship is associated with caregivers’ coping strategies. We hypothesised that Attachment Closeness would be associated with adaptive coping (Positive Attitude, Social Support, Problem Orientation), whereas Attachment Conflict would be associated with maladaptive coping (Avoidance). We conducted multiple linear regression models, adjusted for key covariates and with robust standard errors clustered at the family level, to test these hypotheses. Results: Higher levels of emotional closeness (CPRS) were significantly associated with greater use of adaptive coping strategies, specifically Positive Attitude (β = 0.20, *p* = 0.049) and Problem Orientation (β = 0.26, *p* = 0.002), even after controlling for sociodemographic factors, social support, and resilience. Conversely, higher levels of relational conflict were significantly associated with greater use of the maladaptive Avoidance strategy (β = 0.14, *p* = 0.015). The hypothesis linking closeness to Social Support seeking was not supported. Conclusions: The findings suggest that the parent–child attachment relationship is a significant correlate of caregiver coping strategies in caregivers of children with cancer. Interventions aimed at supporting the caregiver–child dyad by fostering emotional closeness and reducing conflict may promote more adaptive parental coping mechanisms, thereby enhancing family resilience and psychological adjustment throughout the treatment journey.

## 1. Introduction

A diagnosis of paediatric cancer is one of the most stressful events a family can experience. It disrupts daily life and affects the emotional, social, and psychological functioning of the entire family system [1]. Caregivers must cope with intense and prolonged stress while supporting their child throughout demanding medical treatments. During this process, many parents experience high levels of psychological distress, including symptoms of anxiety, depression, and post-traumatic stress [2,3]. For this reason, understanding how caregivers cope with these challenges is essential. Caregiver coping has important implications not only for parental mental health but also for the child’s psychological adjustment and overall family functioning [4,5]. Consequently, caregiver well-being is increasingly recognized as a key component of quality care in paediatric oncology [6].

Coping refers to the cognitive and behavioural efforts individuals use to manage demands that exceed their available resources [7]. Research in paediatric psycho-oncology has consistently shown that coping strategies play an important role in caregiver adjustment [8]. Although coping strategies are not inherently adaptive or maladaptive, certain approaches are generally associated with better psychological outcomes. For example, strategies such as problem solving, positive reframing, and seeking social support are often linked to lower levels of distress and better quality of life [9,10]. In contrast, avoidant strategies such as denial or disengagement are typically associated with poorer psychological outcomes, including higher levels of anxiety, prolonged grief, and post-traumatic stress symptoms [11,12]. However, these strategies may sometimes provide short-term emotional protection in highly stressful situations.

Previous studies have mainly focused on demographic and social factors associated with caregiver coping, such as socioeconomic status, gender, and the availability of social support networks [13]. In comparison, relational factors within the family have received less attention. Attachment theory provides a useful framework for understanding how close relationships influence emotional regulation and responses to stress [14]. The parent–child relationship represents the primary attachment bond for children, but a cancer diagnosis and its treatment may place significant strain on this relationship from the caregiver’s perspective [15].

A secure and emotionally supportive relationship between parent and child—characterized by warmth, trust, and a sense of safety—may function as an important protective factor during illness. Such relationships may help caregivers regulate distress and adopt more adaptive coping strategies by promoting a sense of meaning and efficacy in the caregiving role [16]. In contrast, relationships characterized by conflict, tension, or emotional distance may become an additional source of stress, potentially weakening coping resources and increasing reliance on maladaptive coping strategies such as avoidance [17].

Recent research has begun to highlight the importance of family and relational factors in the psychological adjustment of families facing paediatric cancer. For example, studies have shown that higher levels of family cohesion and emotional expressiveness are associated with lower parental distress [18]. Other research has demonstrated that the quality of the parent–child relationship may influence post-traumatic outcomes in both parents and children [19], as well as broader patterns of coping within the family [20]. Furthermore, a recent review by Santacroce and colleagues [21] emphasized the need for further research examining how family relationships influence health and psychological outcomes in paediatric oncology populations.

Although previous studies have explored the role of caregivers’ individual resources—such as resilience and perceived social support—in shaping psychological adjustment, less is known about how these factors interact with the quality of the parent–child relationship in influencing coping processes. In particular, few studies have directly examined whether the perceived quality of the parent–child relationship independently contributes to caregiver coping when other psychological resources are taken into account [22].

The present study aimed to examine whether the perceived quality of the parent–child relationship is associated with the coping strategies used by caregivers of children with cancer. Specifically, we focused on two relational dimensions measured by the Child–Parent Relationship Scale (CPRS): Closeness, reflecting warmth and emotional connection, and Conflict, reflecting negative and discordant interactions. We investigated whether these relational characteristics function as protective or risk factors in caregivers’ coping responses to illness-related stress. This study makes several contributions to the existing literature. First, it focuses specifically on the dyadic parent–child relationship rather than broader measures of family functioning. Second, the analyses account for the non-independence of observations when data from both parents are included by using family-level clustered standard errors. Third, the models simultaneously control for individual psychological resources (resilience) and social resources (perceived social support), allowing us to examine the unique contribution of the parent–child relationship to caregiver coping.

Based on attachment theory and previous research, we formulated the following hypotheses:

**H1.**  *Higher levels of attachment closeness will be associated with greater use of Positive Attitude coping.*

Rationale: When caregivers perceive their relationship with the child as emotionally close, trusting, and secure, they experience greater confidence in their caregiving role and a stronger sense of emotional connection. This perception supports affect regulation and promotes meaning-oriented coping, such as positive reframing and acceptance. Evidence indicates that parents who appraise their relationship with the child as harmonious and supportive show higher optimism and more constructive emotion regulation when facing paediatric illness [23,24].

**H2.**  *Higher levels of attachment conflict will be associated with greater use of Avoidance coping.*

Rationale: Conversely, when caregivers perceive the relationship with the child as conflictual, tense, or emotionally distant, the caregiving context itself becomes an additional source of stress. Such relational strain may undermine emotional regulation and deplete the psychological resources needed for active coping, increasing the likelihood of avoidant strategies (e.g., denial, mental disengagement). This is consistent with research showing that negative parent–child relationship appraisals are linked to higher experiential avoidance and that parental relational stress is associated with avoidant coping in paediatric illness contexts [25,26].

**H3.**  *Higher levels of attachment closeness will be associated with greater use of Social Support coping.*

Rationale: Bowlby proposed that secure attachment fosters confidence in the responsiveness of others. We hypothesize that the positive relational model experienced within the parent–child dyad generalizes outward, making caregivers who feel connected to their child more likely to believe that others (partners, family, friends, healthcare providers) will be supportive and responsive to their needs. Thus, they may be more inclined to actively seek social support, though support-seeking is also influenced by personality factors such as extraversion. This is underpinned by evidence that attachment security in close relationships is a foundation for effective support-seeking [27], though this specific pathway has been less explored in the paediatric cancer caregiving context.

**H4.**  *Higher levels of attachment closeness will be associated with greater use of Problem Orientation coping.*

Rationale: Attachment theory also describes the “secure base” function, from which an individual feels confident to explore challenges and solve problems. We posit that a secure, close bond with the child provides the emotional foundation that frees up cognitive capacity for active, problem-focused efforts. Caregivers who are not consumed by relational anxieties can better mobilize resources, plan, and take concrete steps to manage treatment demands. This is consistent with studies finding that secure attachment is associated with more adaptive and persistent problem-solving under stress [28] and that family cohesion (a related construct) facilitates active coping in parents of children with chronic conditions [16].

These hypotheses are grounded in the assumption that close and supportive relationships can serve as a “secure base” that facilitates emotional regulation and problem solving under stress. In contrast, conflictual relationships may undermine coping resources and increase the likelihood of disengagement strategies.

By testing these hypotheses, this study aims to clarify whether the perceived quality of the parent–child relationship represents an important relational factor associated with caregiver coping in paediatric oncology.

## 2. Materials and Methods

### 2.1. Study Design and Participants

We conducted a multicentre, cross-sectional exploratory survey between July 2023 and February 2025. We recruited participants from the Paediatric Oncohaematology departments of three Italian hospitals: Fondazione IRCCS Policlinico San Matteo in Pavia, SS. Annunziata Hospital in Taranto, and San Gerardo Hospital in Monza.

Eligible caregivers were parents or primary legal guardians of a child or adolescent aged between 3 and 17 years, in accordance with the protocol approved by the local Ethics Committee. A trained research assistant or psychologist—who was either a hospital staff member or a researcher affiliated with the collaborating university—approached potential participants in the clinic or day hospital setting, provided verbal and written information about the study, and obtained written informed consent. To minimize potential perceived coercion associated with recruitment in clinical settings, participants were explicitly informed that their decision to participate or decline would not affect their child’s medical care in any way. Participation was entirely voluntary, and caregivers were reassured that refusal would have no consequences for their relationship with healthcare providers or for the care received by their child. All recruiters had access to medical records for the purpose of extracting clinical data (child age, diagnosis, treatment phase, relapse status). They extracted these data directly from electronic or paper medical records following a standardized protocol, rather than relying on caregiver reports, to maximize accuracy. Sociodemographic data (caregiver education, occupation) were obtained through brief verbal confirmation from the caregiver.

Only caregivers completed the self-report measures described below. For children aged 7 years and older, age-appropriate verbal assent was obtained after a brief explanation of the study; however, children did not complete any questionnaires. Clinical and demographic data about the child (e.g., age, diagnosis, treatment phase) were extracted from medical records.

After providing written informed consent, participants completed a paper-and-pencil questionnaire battery in a private room within the hospital. Each caregiver completed the measures independently, without the presence of their child or the other parent, to ensure confidentiality and minimize social desirability bias. A research assistant remained available nearby to answer any procedural questions, but did not observe responses.

We collected data during active treatment, excluding the first week post-diagnosis, to avoid acute crisis periods. We used a standardized protocol and identical materials across all three centers to ensure consistency in recruitment and assessment procedures.

The study was conducted in accordance with the Declaration of Helsinki and approved by the S. Matteo Hospital Ethics Committees (Fascicolo 2022-3.11/780; Prot. 0003028/23; Prot. Avvio 0006211/23; Monza: ID Prot. 4125_20.03.24_Mi del 20 March 2024; Taranto: Prot. 429 del 25 July 2023). All participants provided written informed consent.

Participants were informed that their decision regarding participation would not influence the clinical care received by their child.

### 2.2. Measures

Child–Parent Relationship Scale (CPRS). To assess the primary independent variable—the perceived quality of the parent–child relationship—we used the Child–Parent Relationship Scale [15]. This 30-item self-report instrument measures two key dimensions on a 5-point Likert scale: Closeness (e.g., warmth, affection, open communication; 12 items) and Conflict (e.g., negativity, discordant interactions; 12 items), with higher scores indicating higher levels of each dimension. The CPRS has demonstrated good reliability and validity [15] with confirmed factorial structure and construct validity. In this sample, Cronbach’s α was 0.78 for Closeness and 0.85 for Conflict.

Coping Orientation to Problem Experienced-New Italian Version (COPE-NVI). To assess coping strategies—the primary outcome—we used the COPE-NVI [16]. This 60-item questionnaire assesses five coping dimensions on a 4-point Likert scale. We administered the full COPE-NVI to all participants. For this study, we focused on four dimensions: Positive Attitude (PA, 12 items; Cronbach’s α = 0.58), Problem Orientation (PO, 12 items; Cronbach’s α = 0.66), Social Support (SS, 12 items; Cronbach’s α = 0.64), and Avoidance (AS, 12 items; Cronbach’s α = 0.67). Higher scores indicate a greater tendency to use that specific strategy. Although the Positive Attitude (α = 0.58) and Social Support (α = 0.64) subscales demonstrated suboptimal internal consistency in this sample, we retained them for analysis because: (a) the COPE-NVI is the most widely used and validated coping measure in the Italian population; (b) these alpha values are comparable to those reported in other clinical studies using this instrument in high-stress contexts; and (c) exclusion of these theoretically important dimensions would have limited our ability to test the full theoretical model. Nevertheless, we interpret findings involving these dimensions cautiously. We did not include the fifth dimension (Transcendent Orientation) in the main analyses because it was not part of the predefined theoretical model linking parent–child relationship quality to caregiving coping strategies; restricting the analyses to the four targeted dimensions also reduced the number of statistical tests and the risk of Type I error.

It is important to note that the CPRS assesses caregivers’ perceptions of the parent–child relationship quality (closeness and conflict) rather than providing a direct assessment of attachment security as would be measured by observational paradigms (e.g., Strange Situation Procedure) or child attachment narratives. Throughout this manuscript, we use the term ‘attachment relationship’ to refer to this perceived relational quality, consistent with Pianta’s conceptualization of the CPRS as a measure of the parent’s representation of the relationship.

Covariates. To assess perceived social support, we used the Multidimensional Scale of Perceived Social Support (MSPSS) [17,18], a 12-item scale (7-point Likert) assessing support from family (Cronbach’s α = 0.90), friends (Cronbach’s α = 0.95), and significant others (Cronbach’s α = 0.89). To assess resilience, we used the Resilience Scale-14 (RS-14) [19], a 14-item measure (7-point Likert) of the ability to adapt to adversity (Cronbach’s α = 0.84). We collected sociodemographic and clinical data—including caregiver age and gender, child age, diagnosis, treatment phase, and relapse status—using a standardized study-specific form completed by the recruiting researcher. The following clinical variables were extracted from medical records: child age, diagnosis (categorized as leukemias, lymphomas, solid tumors, or other blood diseases), treatment phase (categorized as onset, active phase, or remission), and relapse status (yes/no). Due to inconsistent documentation across centers and time points, we were unable to reliably extract data on treatment intensity (e.g., chemotherapy protocols, radiation dosage), prognostic indicators (e.g., risk stratification), or precise time since diagnosis in days/weeks. We selected the treatment phase as the primary clinical covariate because it represents a clinically meaningful and consistently documented marker of the treatment trajectory that is conceptually relevant to both relational dynamics and coping demands.

### 2.3. Statistical Analysis

We analysed data using Stata 19.5 (StataCorp, College Station, TX, USA). We used descriptive statistics to characterize the sample. We explored initial associations between attachment dimensions (Closeness, Conflict) and coping strategies using bivariable correlations (Pearson’s r derived from generalized linear model residuals).

To test the primary hypotheses and control for Type I error inflation, we pre-specified a hierarchical (gatekeeping) testing procedure [20]. We tested hypotheses in the sequence H1 → H2 → H3 → H4. If a hypothesis yielded a non-significant result at α = 0.05, we treated all subsequent analyses as exploratory and hypothesis-generating rather than confirmatory. This procedure maintains the family-wise Type I error rate at α = 0.05 for the confirmatory hypothesis chain.

We fitted generalized linear models for the association of each coping strategy with attachment dimensions. We adjusted models for caregiver age and gender, child age, treatment phase, social support (MSPSS total score), and resilience (RS-14 total score). All regression models used Huber–White robust standard errors clustered at the family level to account for the non-independence of observations from caregivers belonging to the same family. We used listwise deletion for missing data, which was minimal (<2%).

## 3. Results

### 3.1. Sample Descriptive

Of the 172 eligible caregivers approached during the recruitment period, 165 agreed to participate, yielding a participation rate of 95.9%. The seven caregivers who declined cited time constraints (*n* = 4) or emotional distress (*n* = 3) as reasons for non-participation. The final sample comprised 165 caregivers (88 females, 53%; 77 males, 47%) of 91 paediatric patients. Of these, 74 families (81.3%) contributed data from both parents, whereas 17 families (18.7%) contributed data from a single parent. Notably, fathers represented 47% of the sample, a higher proportion than typically reported in pediatric psycho-oncology studies, likely reflecting recruitment during day-hospital visits where both parents were present. Most participants were recruited from Pavia (*n* = 111, 67.3%), followed by Taranto (*n* = 38, 23.0%) and Monza (*n* = 13, 7.9%). The mean age of caregivers was 43.1 years (SD = 7.5; range: 18–61 years), and the mean age of children was 9.2 years (SD = 4.8; range: 3–17 years). Information on the sample of parents and children is summarised in Table 1 and Table 2.

### 3.2. Bivariable Correlations

Bivariate correlations confirmed significant associations between attachment dimensions and coping strategies (Table 3). Closeness was positively correlated with Positive Attitude (r = 0.31, *p* < 0.001), Social Support (r = 0.18, *p* = 0.02), and Problem Orientation (r = 0.29, *p* < 0.001). Conflict was positively correlated with Avoidance (r = 0.27, *p* = 0.001).

### 3.3. Regression Analyses

H1: Closeness and Positive Attitude. The initial univariable regression analysis revealed a statistically significant positive relationship between attachment closeness and the use of a Positive Attitude coping style (β = 0.28, 95% CI [0.11, 0.46], *p* = 0.002). After adjusting for caregiver age and gender, child age, treatment phase, perceived social support (MSPSS), and resilience (RS-14), the association remained statistically significant (β = 0.20, 95% CI [0.00, 0.39], *p* = 0.049).
H2: Conflict and Avoidance. Supporting our second hypothesis, univariable regression confirmed a significant positive association between parent–child relationship conflict and the use of Avoidance coping strategies (β = 0.15, 95% CI [0.05, 0.24], *p* = 0.003). This relationship held in the multiple regression model after incorporating the same set of covariates (β = 0.14, 95% CI [0.03, 0.25], *p* = 0.015).

As per the pre-specified hierarchical testing plan, the confirmation of H2 allowed for the subsequent testing of H3.

H3: Closeness and Social Support. The univariable analysis for the third hypothesis indicated a positive association between closeness and seeking social support that approached, but did not meet, the threshold for statistical significance (β = 0.22, 95% CI [−0.01, 0.46], *p* = 0.063). However, when we adjusted the model for covariates—most notably the robust effect of general perceived social support (MSPSS)—the association was substantially attenuated and became non-significant (β = 0.11, 95% CI [−0.14, 0.37], *p* = 0.377). Consequently, H3 was not supported.

Following the hierarchical testing protocol, we considered H4 exploratory to provide a more complete picture of the data, with the results interpreted cautiously as hypothesis-generating.

Exploratory Analysis for H4: Closeness and Problem Orientation. Univariable analysis revealed a strong and highly significant positive relationship between closeness and Problem Orientation coping (β = 0.27, 95% CI [0.12, 0.41]). In the multiple regression model controlling for all covariates, the effect size remained virtually unchanged (β = 0.26, 95% CI [0.10, 0.42]) (see Table 4).

### 3.4. Exploratory Analysis by Caregiver Gender

Post hoc exploratory analyses were conducted to examine potential differences between mothers and fathers in attachment dimensions and coping strategies. Independent-samples *t*-tests and exploratory regression models stratified by caregiver gender were performed.

Mothers reported slightly higher levels of emotional closeness compared to fathers, whereas fathers showed marginally higher scores on Problem Orientation coping. However, these differences did not reach statistical significance after adjustment for multiple comparisons.

Moreover, the associations between attachment dimensions and coping strategies remained directionally consistent across genders. In both mothers and fathers, higher closeness was associated with greater use of adaptive coping strategies, whereas higher conflict was associated with greater use of avoidance.

No significant interaction effects between caregiver gender and attachment dimensions were observed in relation to coping outcomes.

Given the exploratory nature of these analyses and the limited statistical power for subgroup comparisons, these findings should be interpreted cautiously as hypothesis-generating and warrant replication in larger, pre-registered studies.

## 4. Discussion

This study provides consistent evidence that the quality of the parent–child attachment relationship is significantly and independently associated with coping strategies among caregivers of children with cancer, even after controlling for key psychological resources like general social support and resilience. Our findings confirm that emotional closeness is associated with adaptive, engagement-focused coping, while relational conflict is associated with maladaptive avoidance, thereby painting a more nuanced picture of family resilience in paediatric psycho-oncology [29]. These findings highlight the value of adopting a dyadic, attachment-informed lens in pediatric psycho-oncology—a field traditionally focused on individual caregiver factors—by demonstrating that the parent–child relational bond independently shapes coping processes.

Support for H1 and the exploratory finding for H4 indicate that perceiving a close bond with the child provides caregivers with an emotional foundation for constructive engagement with the illness. This manifests not as passive optimism, but as an active orientation characterized by acceptance, positive reframing, and pragmatic problem-solving. This result suggests that the emotional quality of the parent–child bond is a unique and independent contributor to a caregiver’s ability to maintain a constructive and accepting outlook in the face of their child’s illness, above and beyond their general resilience or external social support. Thus, H1 was supported. This aligns powerfully with Bowlby’s concept of the “secure base” [11], which allows an individual to explore challenges and manage stress from a position of safety. Our results empirically extend this concept to the parent in the caregiving dyad, suggesting that feeling connected to and effective with their child becomes a core source of strength and agency [30]. This emotional security may buffer against helplessness, enabling caregivers to confront the immense challenges of treatment with more adaptive cognitive and behavioural strategies, a finding that resonates with recent work on the role of parental self-efficacy in paediatric illness adjustment [31,32]. However, while this association was statistically significant, the modest reliability of the Positive Attitude subscale suggests that this finding should be interpreted with some caution pending replication.

The confirmation of H2 (Conflict → Avoidance) underscores the potent, detrimental impact of a conflictual relationship. Caregivers who perceive their interactions with their ill child as negative and discordant are significantly more likely to engage in avoidant coping, such as denial and behavioural disengagement. The consistency of the beta coefficient across models suggests that relational conflict is reliably associated with avoidant coping strategies. This finding underscores that relational discord functions as a specific stressor that promotes cognitive and behavioural disengagement, independent of the caregiver’s demographic background or other psychological resources. Therefore, H2 was supported.

This finding is consistent with models of relational stress and allostatic load, where chronic interpersonal conflict acts as a toxic stressor that overwhelms emotional and cognitive regulatory capacities [33,34]. Avoidance, in this context, can be understood as a protective—though ultimately dysfunctional—short-term strategy to escape the compounded distress of the illness and a strained relationship [35]. This is critically important, as avoidant coping is a well-established transdiagnostic risk factor for longer-term psychological morbidity, including anxiety, depression, and post-traumatic stress symptoms in caregivers [12,36].

The rejection of H3 (Closeness → Social Support) is an important and clarifying finding that refines, rather than contradicts, attachment theory. Attachment theory posits that secure relationships foster internal working models of others as responsive and supportive [14]. However, our findings suggest a more nuanced, domain-specific pathway: the parent–child bond may regulate internal coping processes (affect regulation, cognitive reframing, problem-solving) more directly than it governs outward help-seeking behavior. This distinction is theoretically coherent: seeking social support requires not only the expectation that others will be responsive (an internal working model) but also the perceived availability of support networks, opportunities for support-seeking in the hospital environment, and personality traits such as extraversion [37,38]. The parent–child bond, while foundational to the caregiver’s sense of security, may be too specific a relational factor to directly predict the activation of external support systems, which are contingent on a separate set of environmental and interpersonal resources [39]. Indeed, the robust effect of general perceived social support (MSPSS) in our models—which attenuated the closeness–support association to non-significance—suggests that caregivers’ global perceptions of support availability, rather than the specific parent–child bond, may be the more proximal predictor of active support-seeking behavior. Thus, our findings refine attachment theory by suggesting that different facets of the attachment system (specific dyadic bonds vs. general representations of support) may have differentiated effects on distinct coping domains.

Furthermore, our cross-sectional design precludes causal inference and cannot rule out plausible alternative explanations. For instance, it is possible that a caregiver’s mental health status—such as clinically significant depression or anxiety—could negatively bias their perception of the parent–child relationship while simultaneously driving greater reliance on avoidant coping strategies. These and other third-variable influences highlight the need for longitudinal research to disentangle the temporal and transactional dynamics between relational appraisals, coping, and psychological distress across the treatment trajectory.

### 4.1. Clinical Implications

These findings suggest potential avenues for clinical intervention that warrant future investigation. If replicated in longitudinal studies, the observed associations raise the possibility that supporting the parent–child relationship could contribute to healthier caregiver coping. Brief assessment of parent–child relationship quality (e.g., using measures of closeness and conflict) might help identify caregivers experiencing relational strain who could benefit from additional support [40].

However, it is important to emphasize that our cross-sectional data do not provide empirical support for any specific intervention. As a speculative direction for future research, interventions originally developed for other populations—such as Attachment and Biobehavioral Catch-up (ABC) [41], Attachment-Based Family Therapy (ABFT) [42], Parent–Child Interaction Therapy (PCIT) [43], or Video-feedback Intervention to Promote Positive Parenting and Sensitive Discipline (VIPP-SD) [44]—could hypothetically be adapted and piloted in pediatric oncology settings to evaluate their feasibility and preliminary impact on coping and distress. Rigorous testing through randomized controlled trials would be necessary to determine whether such approaches are effective in this population. The goal of such future research would be to examine whether strengthening bonds of warmth and security (closeness) and reducing negative interaction patterns (conflict) might indirectly support healthier caregiver coping [45,46].

### 4.2. Strengths and Limitations

The strengths of this study include its multicentre design, which increased the geographic and clinical heterogeneity of the sample; a relatively large sample size for a clinical population; the use of well-validated instruments; and a robust statistical approach that controlled for key confounding variables and accounted for the non-independence of caregiver observations by clustering robust standard errors at the family level.

Several limitations must be acknowledged. The cross-sectional design precludes any causal inference and prevents us from determining the direction of effects; it is possible that coping strategies influence the perception of the relationship, that the relationship quality shapes coping, or that a third variable influences both. Longitudinal studies are needed to examine the temporal and potentially transactional relationships between attachment dynamics and coping across the treatment trajectory and into survivorship [47].

The involvement of hospital-employed psychologists in data collection, while ensuring clinical sensitivity and access, may have introduced social desirability bias, as participants might have been reluctant to disclose negative perceptions of their relationship with their child to a familiar healthcare provider. Future studies should consider using independent data collectors not directly involved in clinical care to minimize this potential bias.

The sample was recruited from only three Italian centres, which, despite being multicentre, may limit the generalizability of findings to other cultural contexts with different family structures and support systems [48]. Furthermore, the study relied solely on caregiver self-report, which may be subject to social desirability bias and shared method variance.

An important alternative explanation that we could not rule out is the potential role of caregiver psychological distress—particularly depression and anxiety—as a third variable influencing both the perception of the parent–child relationship and coping strategies. Depressive symptoms are associated with negative cognitive biases that could lead caregivers to perceive their relationship with their child more negatively (reporting lower closeness and higher conflict) while simultaneously endorsing more maladaptive coping strategies (higher avoidance) and underreporting adaptive coping [49,50]. Although we controlled for resilience, which is inversely related to depression, we did not directly assess depressive or anxiety symptoms. Thus, unmeasured psychological distress could confound the observed associations. This is particularly relevant given the shared method variance inherent in self-report designs: all measures were completed by the same informant at the same time point, which can inflate associations due to common method bias [51]. Other plausible third variables include caregiver personality traits (e.g., neuroticism, which is associated with both relational conflict and avoidant coping) [52], family socioeconomic resources (which we partially assessed but may not have fully captured), and the child’s own adjustment and behavior, which could influence both the parent–child relationship and parental coping [53]. Longitudinal studies with repeated measures are needed to disentangle the temporal and potentially bidirectional relationships among these variables. Multi-method approaches incorporating observational measures of parent–child interaction, clinician-rated distress, and child reports would help address shared method variance and provide a more rigorous test of causal hypotheses [54].

An additional limitation concerns the internal consistency of some COPE-NVI subscales. In the present sample, the Positive Attitude (α = 0.58) and Social Support (α = 0.64) subscales showed lower-than-optimal reliability. Although these values are comparable to those reported in previous clinical studies using the COPE-NVI in high-stress contexts, they indicate increased measurement error, which likely attenuates observed associations and increases the risk of Type II error. Consequently, the significant association we observed for Positive Attitude may represent an underestimation of the true effect size, while the non-significant finding for Social Support may reflect insufficient reliability rather than a true absence of association. Findings involving these dimensions should therefore be interpreted with particular caution and warrant replication using instruments with stronger psychometric properties or after further validation of the COPE-NVI in pediatric oncology populations.

The high proportion of fathers, while a strength in terms of representation, may reflect specific recruitment conditions (e.g., day-hospital setting) and limit direct comparability with studies where maternal samples predominate. Furthermore, this study relied solely on caregiver-reported perceptions of the parent–child relationship. Future research should incorporate multi-informant data, including child self-report (where developmentally appropriate) and observational measures, to capture a more complete and objective picture of attachment processes [54].

Although we included the treatment phase as a covariate in all regression models, we did not systematically include more fine-grained clinical variables such as diagnosis type, treatment intensity, prognosis, and time since diagnosis as covariates. This was due to incomplete availability of harmonized clinical data across centers and limited statistical power for reliable subgroup analyses. As a result, residual clinical confounding cannot be fully excluded and should be addressed in future studies with larger samples and more detailed longitudinal clinical profiling.

The analytical strategy adopted in this study reflects the standards and procedures commonly applied in hospital-based clinical research. We conducted the study within structured institutional research frameworks, following standardized protocols and in close collaboration with hospital epidemiology and biostatistics units. The selection of covariates, the handling of clustered data, and the overall statistical approach were informed by established clinical research guidelines. However, we did not formally pre-register the study, and we developed the analytical plan within the institutional approval process rather than through a public registration platform. While this approach is consistent with many applied clinical studies conducted in hospital settings, it may increase the risk of selective reporting and limit full transparency. Therefore, future research would benefit from prospective pre-registration and replication in independent samples to further strengthen the robustness of these findings.

Furthermore, this study relied solely on caregiver-reported perceptions of the parent–child relationship. The exclusive use of caregiver self-report introduces mono-informant bias, which may inflate associations due to shared method variance—that is, caregivers who report high closeness may be dispositionally inclined to report positive experiences across measures. Conversely, caregivers experiencing psychological distress may negatively bias their perceptions of both the relationship and their coping, potentially confounding the observed associations. Future research should incorporate multi-informant data, including child self-report (where developmentally appropriate) and observational measures, to capture a more complete and objective picture of attachment processes [54].

### 4.3. Future Directions

Building on this work, future research should adopt longitudinal designs to track how attachment dynamics and coping strategies co-evolve and influence each other over time, particularly at critical junctures like diagnosis, treatment completion, and relapse. A key next step would be to investigate whether coping strategies mediate the relationship between parent–child attachment quality and critical caregiver mental health outcomes (e.g., clinical levels of anxiety, depression, or PTSD) [55]. Finally, the most crucial avenue for applied clinical research is the development and testing of the efficacy of brief, targeted, and manualized interventions specifically designed to enhance attachment closeness and reduce conflict in families facing paediatric cancer, and to evaluate their impact on coping and long-term psychological adjustment [56].

## 5. Conclusions

In conclusion, this study identifies the parent–child relationship as an important factor associated with coping strategies among caregivers of children with cancer. These findings suggest that attending to the parent–child relationship may offer an additional avenue for supporting caregiver coping, complementing existing individual-focused approaches. Consequently, if future longitudinal research confirms these associations, attachment-informed screening and interventions could represent a promising preventive strategy to support family resilience throughout the cancer journey.

## Figures and Tables

**Table 1:** Caregiver characteristics.

Caregivers’ Characteristics	Value (%)
Mean age	43.1 ± 7.5
Marital Status	
Single	3 (1.8%)
Married/civil union	149 (90.3%)
Separated/divorced	8 (4.8%)
Widowed	5 (3%)
Education	
High school diploma	71 (43%)
PhD/Specialization	5 (3%)
University degree	35 (21.2%)
Middle school certificate	54 (32.7%)
Current Occupation	
Unemployed/Inactive	31 (18.8%)
Plant & Machine Operators/Agricultural & Industrial Workers	26 (15.8%)
Clerical support Workers	23 (13.9%)
Service and Sales Workers	16 (9.7%)
Technicians and Associate Professionals	16 (9.7%)
Skilled Trades Workers	13 (7.9%)
Professionals	11 (6.7%)
Armed Forces	7 (4.2%)
Managers	4 (2.4%)
Elementary Occupations	1 (0.6%)
Other	17 (10.3%)

**Table 2:** Child characteristics.

Children’s Characteristics	Value (%)
Mean age	9.76
Diagnosis	
Leukemias	48 (52.7%)
Lymphomas	8 (8.8%)
Solid Tumors	12 (13.2%)
Other blood diseases	23 (25.3%)
Treatment Status	
Onset	24 (26.4%)
Active phase	53 (58.2%)
Remission	14 (15.4%)
Relapse	
No	80 (87.9%)
Yes	11 (12.1%)
Treatment Phase	
Chemotherapy	54 (59.3%)
Maintenance	8 (8.8%)
Immunotherapy	29 (31.9%)

**Table 3:** Bivariate correlations between attachment dimensions and coping strategies.

Variable	1	2	3	4	5	6
1. CPRS Closeness	—					
2. CPRS Conflict	−0.42 ***	—				
3. COPE Positive Attitude	0.31 ***	−0.16 *	—			
4. COPE Avoidance	−0.11	0.27 **	0.05	—		
5. COPE Social Support	0.18 *	−0.02	0.44 ***	0.23 **	—	
6. COPE Problem Orientation	0.29 ***	−0.17 *	0.71 ***	0.09	0.52 ***	—

* *p* < 0.05, ** *p* < 0.01, *** *p* < 0.001.

**Table 4:** Multiple regression models examining associations between attachment dimensions and coping strategies.

Dependent Variable	Variable	β	95% CI	*p*-Value	Adjusted R^2^
Positive Attitude	CPRS Closeness	0.20	[0.00, 0.39]	0.049	0.32
Avoidance	CPRS Conflict	0.14	[0.03, 0.25]	0.015	0.18
Problem Orientation	CPRS Closeness	0.26	[0.10, 0.42]	0.002	0.45

Note: *n* = 165 caregivers. Models adjusted for caregiver age, gender, child age, treatment phase, MSPSS and RS-14.

## Data Availability

Data are unavailable due to privacy restrictions.
